# The Discoverer of Anæsthesia

**Published:** 1873-07

**Authors:** 


*The Discoverer of Anæsthesia.*- The following appears in the June 21st number of the *Medical and Surgical Reporter*, which has always earnestly advocated and defended the just claims of Dr. Horace Wells, a practicing dentist:—

The eleventh day of December, 1844, was an era, and a very important one, in the history of surgery. On that day Horace Wells, of Hartford, Conn., for the first time made practical demonstrations of the application of anæsthetics for the purpose of subduing pain under surgical operations. While under the influence of nitrous oxide gas, he had a sound tooth extracted. He remained under the influence of the gas some time after, and immediately upon recovering from it threw up his arms and exclaimed, " A new era in tooth pulling ! It did not hurt me more than the prick of a pin. It is the greatest discovery ever made !" From this time the principle of anæsthesia became an established one in surgery, and by degrees came into general use. Wells pursued his experiments with nitrous oxide, ether, and other agents, with an enthusiasm which eventually cost him his life. Finding that others were seeking to rob him of the credit of his great discovery, he became disgusted, disappointed, and dispirited. He then went to New York to lay his claims as the discoverer of anæsthesia before the profession of the great metropolis. Soon after his arrival there he manifested symptoms of mental aberration, and on the 24th of January, 1848, in a fit of madness, ended his life with his own hands. He thus left his family unprovided for, and an open field for the unscrupulous to poach upon to rob him of his well-earned honors. To the discredit of the medical profession, many of them were for a time led astray by the specious representations of these parties. But the sober second thought of the profession has become enlisted on behalf of the memory of the unfortunate Wells, and such men as the late Sir James Y. Simpson, Storer, Sims, Doremus, Hamilton, Squibb, and many others of the leading minds of the profession, are using their influence to do justice to the memory of the real discoverer of the application of anæsthesia in surgical operations.

Expression was given to these sentiments at a large and enthusiastic meeting in New York on the 21st of May. The meeting was addressed by Drs. Marion Sims, Ogden Doremus, Frank H. Hamilton, and others. We welcome any effort to do justice to the memory of one whose discovery, on the 11th of December, 1844, soon deprived surgical operations of their terror, and proved such a boon to suffering humanity, and such an invaluable aid to the surgeon in the use of surgical instruments. We feel proud of the fact that for twenty-five years the *Medical and Surgical Reporter* has constantly and earnestly advocated and defended the claims of Wells. May they yet receive that full and free recognition at the hands of the public and the general government which they undoubtedly deserve.

In a communication from Dr Henry J. Bigelow, of Boston, published in a New York paper, that gentleman, although his object is to support the claim of Morton, is compelled to admit the priority of Wells' practical application of anæsthesia for surgical purposes, though he endeavors to belittle his achievements, and claims that Wells abandoned the use of anæsthetics.

In reply to this, Dr. G. Q. Colton very emphatically upsets the theory of the Wells' abandonment. "We have," he says, "the sworn testimony of about forty of the most respectable citizens of Hartford, that during the years 1845 and 1846 Wells extracted teeth for them without pain, using the gas as an anæsthetic. He was in constant use of the gas for about eighteen months, when his health gave way, and he went to Europe. Even in Europe he did not abandon his discovery, for he presented his claims to the Academy of Sciences in Paris, and that institution, in recognition of the services, conferred upon him the title of M. D.

"As soon as Wells returned to this country he resumed the use of the gas, and continued it until his death, which occurred on the 24th of January, 1848.

"But he met the most determined and bitter opposition from all quarters. It was at that time too much to believe that the inhalation of so little gas or vapor would destroy the pain of a surgical operation! Dr. Wells did all that a man could do, while he lived, to prove to the world the value of his discovery. Should he be deprived of the honor of the discovery because the public were incredulous and repudiated his claims?

"Wells died before the merits of the gas were generally recognized. After his death Dr. Morton set up the claim that nitrous oxide was not an anæsthetic, and therefore that Wells had discovered nothing! No one had used the gas to produce anæsthesia save Wells, and Morton was enabled to gain a general assent to the position he took, namely, that nitrous oxide not being an anæsthetic, therefore he, Morton, was the discoverer of anæsthesia! If at that time and during the lifetime of Mr. Wells the gas had proved to be what it really is, and what I have demonstrated it to be, the best and safest anæsthetic known, we never should have heard of Morton as the discoverer of anæsthesia.

"When I revived the use of the gas in 1863, I had this general incredulity respecting its powers to contend with. I was met on all sides by the assertion that Wells had tried the gas and it had proved a failure. I expended eight thousand dollars the first year in advertising, advocating, and defending it; and in all this time did not realize a dollar of profit from my business. Is it any wonder that poor Wells, who had no money to spend, should encounter opposition and discouragement in its first introduction?

"It should be remembered that Wells' first experiment, for which I gave him the gas, was on the 11th of December, 1844, and that the first experiment by Morton was on the 30th of September, 1846; also, that Morton was stimulated to this experiment by information derived from Wells, and newspaper notices of Wells' operations.

"In view of all these facts," says Dr. Colton, "how can any one hesitate to award the honor of the discovery of anæsthesia to Dr. Wells?"

